# Prognostic Impact of Right Ventricular Damage Markers on CT in Patients Undergoing TAVI

**DOI:** 10.3390/life15071020

**Published:** 2025-06-26

**Authors:** Veysel Özgür Barış, Özkan Karaca, Murat Harman, Fatih Poyraz, Fatma Köksal, Alperen Öztürk, Emin Erdem Kaya, Murat Kaya, Mehmet Ballı, Mehmet Ali Kobat

**Affiliations:** 1 Cardiology Department, Gaziantep City Hospital, Şahinbey, 27700 Gaziantep, Türkiye; fpoyraz78@gmail.com (F.P.); eerdemky@gmail.com (E.E.K.); 2 Cardiology Department, Mersin City Hospital, Toroslar, 33240 Mersin, Türkiye; mdozkrc@gmail.com (Ö.K.); fatmakoksaldr@gmail.com (F.K.); dr_mehmetballi@hotmail.com (M.B.); 3 Cardiology Department, Fırat University School of Medicine, 23119 Elazığ, Türkiye; mharman20@hotmail.com (M.H.); ozturkalperen.md@gmail.com (A.Ö.); drmehmetalikobat@gmail.com (M.A.K.); 4 Radiology Department Gaziantep City Hospital, Şahinbey, 27700 Gaziantep, Türkiye; drmuratkaya85@gmail.com

**Keywords:** TAVI, CT, mortality, prognostic marker

## Abstract

**Background:** In patients undergoing surgical aortic valve replacement due to severe aortic valve stenosis (AS), the presence of right ventricular damage markers (RVDMs) determined by echocardiography is a poor prognostic indicator. However, such data is not available in patient groups who have undergone transcatheter aortic valve replacement (TAVI). The aim of this study is to investigate the prognostic value of RVDM determined by computed tomography (CT) in a patient group undergoing TAVI. **Material and Method:** This multicentre, prospective study included 184 patients who underwent TAVI. In basal CT, the pulmonary artery (PA) diameter and right ventricular outflow tract myocardial thickness (RVOTMT) in mid-systole and maximal right and left ventricular diameter (RVD/LVD) ratios in mid-diastole were measured as indicators of RVDM before the TAVI procedure. The primary endpoint of the study was 1-year mortality. **Results:** The primary endpoint of the study was observed in 42 patients (22%). Among the CT parameters, the PA diameter, RVOTMT, and maximal RVD/LVD ratio were observed to be higher in the mortality group (*p* < 0.001). In the ROC analysis, a PA diameter of 30.5 and above had a 78% sensitivity and 82% specificity (AUC: 0.87 95% CI 0.82–0.93, *p* < 0.001), an RVOTMT of 4 mm and above had 90% sensitivity and 87% specificity (AUC: 0.93 95% CI 0.87–0.99, *p* < 0.001), and a maximal RVD/LVD ratio of 0.91 and above showed 90% sensitivity and 92% specificity (AUC: 0.94 95% CI 0.89–0.99, *p* < 0.001) to predict one-year mortality. In the Cox regression analysis, the RVOTMT and maximal RVD/ LVD ratios were found to be the best independent parameters showing 1-year mortality. **Conclusion:** This study showed that RVDMs determined by CT are strong predictors of 1-year mortality in severe AS patients treated with TAVI.

## 1. Introduction

Severe aortic valve stenosis (AS) is seen in approximately 12.5% of people over the age of 75 and is the most common valve disease in the western world [1,2]. Current guidelines recommend therapy in symptomatic patients with an aortic valve area of less than 1 cm^2^ and a mean valve gradient greater than 40 mmHg. It can be treated with surgical aortic valve replacement (sAVR) or transcatheter aortic valve implantation (TAVI) [3]. TAVI is recommended for elderly patients over 75 years of age or patients at risk, with a Euroscore 2 score of 8 or higher. The decision-making processes for these treatments do not include anatomical parameters that can determine the prognosis, and cardiac damage in this patient group leads to major deficiencies in treatment guidance.

In the study by Genereux et al. [4], it was shown that mortality was higher in a group with right ventricular dysfunction who underwent sAVR. In the study, only a patient group who underwent sAVR was included, and only echocardiography was used. This trial had several limitations like subjectivity, reproducibility, and inter- and intra-observer variability. Therefore, the data are limited for the patient group that underwent TAVI and there is a level of subjectivity because of using only echocardiography. In this study, we aimed to determine the prognostic value of right ventricular damage markers determined by computed tomography (CT), which is more objective than echocardiography, in patients undergoing TAVI.

## 2. Material and Method

### 2.1. Study Population

This is a prospective, multicentre, observational study conducted in 3 tertiary care centres (Gaziantep City Hospital, Mersin City Hospital, Fırat University) with high procedure volumes in Türkiye.

As inclusion criteria, the study group consisted of patients undergoing TAVI due to severe AS. Exclusion criteria included inadequate pre-procedure CT and echocardiographic images, lack of 1-year follow-up, and previous tricuspid or pulmonary artery surgery.

**Figure 1:**
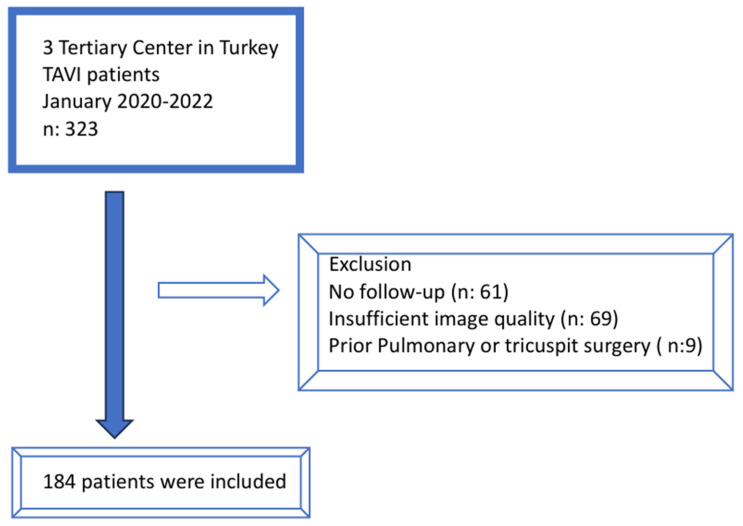
Flowchart of trial.

The data of 323 patients who underwent TAVI for severe symptomatic AS between January 2020 and January 2022 were analysed. Sixty-one patients without a 1-year follow-up, sixty-nine patients with poor-quality pre-procedural tomography, and nine patients who had previously undergone tricuspid valve surgery were excluded from the study (Figure 1). The clinical characteristics, Euroscore 2 scores, and baseline echocardiographic and tomographic measurements of 184 patients were evaluated.

The study protocol was developed in accordance with the Declaration of Helsinki and it was approved by the Gaziantep City Hospital Ethics Committee (date 18.06.2025, no. 228/2025).

### 2.2. Echocardiography

Baseline echocardiographic evaluation of all patients included in the study was performed by physicians specialized in the field using multiple echo machines (Vivid 7, Vivid 9 [GE Medical Systems, Milwaukee, WI, USA]; Epiq7 [Philips Healthcare, Inc., Andover, MA, USA]).

The diagnosis of severe AS was established according to the guidelines via echocardiography (aortic valve area < 1.0 cm^2^, mean aortic valve gradient > 40 mm Hg, jet velocity > 4 m/s) [1].

TR severity was assessed using an integrated approach and graded as none, mild, moderate, or severe according to current guidelines [5]. We defined patients who had moderate or greater TR as “significant TR” and patients who had mild or less TR as “non-significant TR”. RV dimensions were measured at the base in the 4-chamber view. Measurements of ≥42 mm at the base were considered indicative of RV dilation [6]. Systolic pulmonary artery pressure was measured from the tricuspid jet flow [6].

### 2.3. Tomographic Measurements

As recommended, all patients underwent pre-TAVI ECG-gated cardiac CT scans with at least 64 slices [5]. Systolic-phase imaging was used for aortic valve and pulmonary artery diameter measurements, while diastolic-phase imaging was employed for ventricular measurements [7]. CT measurements were made in a core laboratory. An experienced and blinded radiologist and cardiologist manually calculated the markers by using the Sygno.via workstation. The researchers made the measurements without knowledge of the patients’ clinical course and the primary endpoint of the study. Interobserver variability was tested by Kendall’s coefficient of concordance and the test statistic W was calculated as 0.98. Patients with poor tomographic image quality, inadequate for measurement, were excluded from the study.

**Figure 2:**
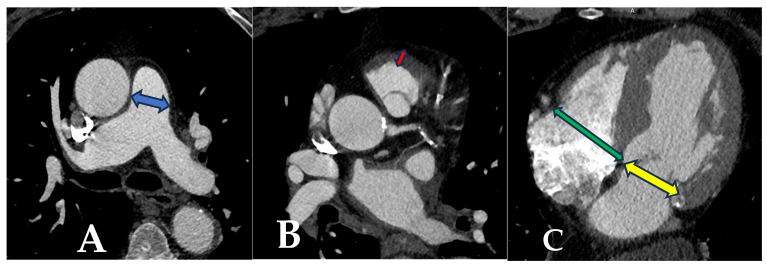
(**A**) Main pulmonary artery diameter was measured perpendicular to the vessel axis at the widest point. (**B**) The muscular thickness of the RVOT was measured anteriorly ~1 cm below the pulmonary valve. (**C**) Apical 4-chamber maximal right ventricular diameter and left ventricular diameter ratio.

The main pulmonary artery (PA) diameter was measured perpendicular to the vessel axis at the widest point (Figure 2A). The muscular thickness of the RVOT was measured anteriorly ~1 cm below the pulmonary valve (Figure 2B) [8].

The apical 4-chamber maximal right ventricular diameter (RVD) and left ventricular diameter (LVD) ratios were considered as indicators of right ventricular dysfunction [9]. Axial images were used to obtain 4-chamber views through predefined methods, and the RVD/LVD ratio was calculated (Figure 2C) [10].

### 2.4. Operative Details

All enrolled patients underwent TAVI using the CoreValve or Evolut R (Medtronic, Minneapolis, MN, USA), Myval (Meril Lifesciences, Vapi, Gujarat, India), Accurate Neo (Boston Scientific, Marlborough, MA, USA), and Portico (Abbott Cardiovascuylar, Plymouth, MN, USA) systems. The prosthesis type and size were decided by the local heart team on the basis of findings on preprocedural echocardiography and multidetector computed tomography. The access site for TAVR was selected by the individual heart team.

### 2.5. Endpoint

The primary endpoint of the study was defined as 1-year mortality. One year after the TAVI procedure, all patients or their first-degree relatives were contacted via teleconsultation.

The mortality status of the patients was ascertained, and all surviving patients were invited to the hospital for examination.

The baseline clinical, laboratory, echocardiographic, and tomographic data of patients who died and those who survived after one year were compared.

### 2.6. Statistical Analysis

All statistical analyses were conducted using the SPSS software (version 20.0 for Windows, SPSS Inc., Chicago, IL, USA). The data obtained were compared between groups.

The data of continuous variables that fit the normal distribution are given as mean ± standard deviation (SD) and the comparison was performed by Student’s *t*-test. The data of continuous variables that did not fit the normal distribution are given as median and interquartile range (IQR), and the Mann–Whitney U test was used for comparison. The data for categorical variables are given as percentages and comparison was performed by the chi-square test.

To identify the role of Ct parameters in determining the primary endpoint, receiver operating characteristic (ROC) curves were drawn and the area under the curve was calculated.

A value of *p* < 0.05 was considered statistically significant. Variables found to be statistically significant in univariate analysis were re-evaluated with Cox regression analysis. Kaplan–Meier analysis was performed to determine the survival of these variables.

## 3. Results

A total of 184 patients with complete one-year follow-up data and good pre-procedural CT image quality were included in the analysis. Of these patients, 54.1% were female (*n* = 99) and the mean age was 76.9 ± 7.3 years. Prior to the procedure, the mean aortic valve gradient was 52 ± 10 mmHg. In the patients included in the study, 34 Medtronic (18.4%), 52 Abott (28%), 56 Boston (30.4%), and 42 (22.8%) Meril valves were used. The primary endpoint of the study, one-year mortality, was observed in 42 patients (22%) (Table 1).

When comparing the baseline clinical characteristics between the groups with and without mortality at one year, it was found that the group with mortality was older [81 (9.5) vs. 76 (9) years, *p* = 0.002] and had a higher prevalence of coronary artery disease (CAD) (85.7% vs. 71.8%, *p* = 0.04). The rates of diabetes, hypertension, and chronic obstructive pulmonary disease (COPD) were similar between the two groups.

In terms of pre-procedural laboratory parameters, the group with one-year mortality had higher baseline creatinine levels [1.1 (0.49) vs. 0.95 (0.5) mg/dL, *p* = 0.019] and consequently lower GFR [52 (33) vs. 71 (32) mL/min/1.73 m², *p* < 0.001]. The baseline serum albumin, haemoglobin, and WBC levels were similar in both groups.

Comparing the baseline echocardiographic characteristics, it was observed that the EF was similar in both groups; however, systolic pulmonary artery pressure [45 (33) vs. 40 (27) mmHg, *p* = 0.014], the incidence of severe tricuspid valve regurgitation (TR) (21% vs. 11.9%, *p* = 0.008), and RV dilation (48.6% vs. 22.9%, *p* = 0.03) were higher in the group with mortality.

The Euroscore 2 score was similar in both groups (*p* = 0.155).

The tomographic data showed that the pulmonary artery diameter was more dilated [34.5 (28–43) vs. 25 (15–36), *p* < 0.001] in the group with 1-year mortality (Table 1).

**Figure 3:**
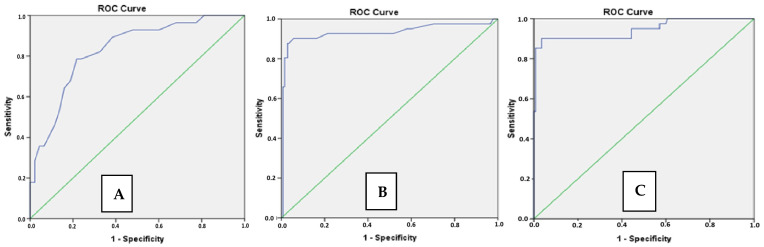
ROC curves representing the relationship between the right ventricular damage markers and one-year mortality: (**A**) Pulmonary artery diameter; (**B**) right ventricular outflow tract thickness; (**C**) maximal right and left ventricular diameter ratios.

The area under the ROC curve, plotted to demonstrate the effectiveness of pulmonary artery diameter in predicting 1-year mortality, was 0.87 [95% confidence interval (95% CI: 0.82–0.93, *p* < 0.001] (Figure 3). Through the ROC curve, when the pulmonary artery diameter was 30.5 mm or higher, the sensitivity was 78% and specificity was 82% in determining in-hospital mortality. Through the ROC curve, categorically, a pulmonary artery diameter of 30.5 mm or higher was found to increase the 1-year mortality by 11 times (OR = 11.6; 95% CI = 4.3–31.2; *p* < 0.001).

The tomographically measured right ventricular outflow tract was thicker [6.4 (4.9–7.2) vs. 3.5 (2.5–4.5), *p* < 0.001] in the group with 1-year mortality (Table 1). The area under the ROC curve, plotted to demonstrate the effectiveness of right ventricular myocardial thickness in predicting 1-year mortality, was 0.93 [95% confidence interval (95% CI: 0.87–0.99, *p* < 0.001] (Figure 3). Through the ROC curve, when the right ventricular myocardial thickness 4 mm was or higher, the sensitivity was 90% and the specificity was 87% in determining in-hospital mortality. Through the ROC curve, categorically, a right ventricular outflow tract thickness of 4 mm or higher was found to increase the 1-year mortality by 30 times (OR = 30; 95% CI = 8.5–106.2; *p* < 0.001).

The apical 4-space maximal RV/LV diameter ratios were higher in the group with 1-year mortality (1.15 ± 0.19 vs. 0.77 ± 0.12, *p* < 0.001) (Table 1). The area under the ROC curve, plotted to demonstrate the effectiveness of the RVD/LVD ratios in predicting 1-year mortality, was 0.94 [95% confidence interval (95% CI: 0.89–0.99, *p* < 0.001] (Figure 3). Through the ROC curve, when the RVD/LVD ratios were 0.91 or higher, the sensitivity was 90% and the specificity was 92% in determining in-hospital mortality. Through the ROC curve, categorically, an RVD/LVD of 0.91 or higher was found to increase the 1-year mortality by 46 times (OR = 46.2; 95% CI = 12.8–167.7; *p* < 0.001).

In the univariate analysis, the variables found to be associated with one-year mortality (age, coronary artery disease, glomerular filtration rate, echocardiographic pulmonary artery pressure, severe tricuspid valve insufficiency, echocardiographic right ventricular dilation, tomographic pulmonary artery diameter, RVOT thickness, RVD/LVD ratio) were entered into a Cox regression as a multivariate analysis. It was determined that a right ventricular myocardial thickness of 4 mm or greater and a maximal RVD/LVD ratio of over 0.91 were independent predictors of 1-year mortality (Table 2). The RVD/LVD ratio was also identified as the most significant independent factor in predicting one-year mortality in patients undergoing TAVI, based on the Wald statistics (Table 2).

**Figure 4:**
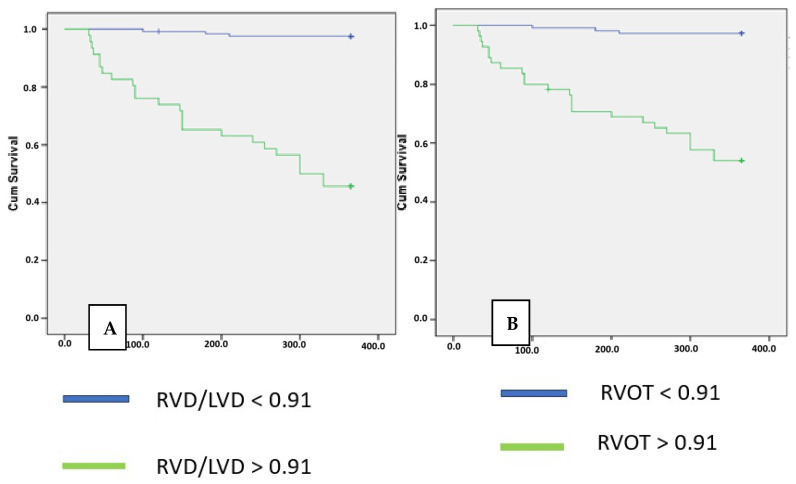
Kaplan–Meier estimates of the percentages of patients with mortality through 12 months as the primary endpoint. Panel (**A**): Maximal right and left ventricular diameter ratio. Panel (**B**): Right ventricular outflow myocardial thickness.

In the Kaplan–Meier analysis, it was demonstrated by the log rank that an RVOT myocardial thickness of 4 mm or greater and maximal RVD/LVD ratios of 0.91 or greater significantly predicted one-year mortality (*p* < 0.001) (Figure 4).

## 4. Discussion

In this multicentre study, the prognostic value of right ventricular cardiac damage markers (pulmonary artery diameter, right ventricular outflow tract thickness, and maximum RV/LV diameter ratios) determined by CT in patients undergoing TAVI was investigated, and it was shown that these markers have significant prognostic value.

A PA diameter of 30.5 and above had 78% sensitivity and 82% specificity (AUC: 0.87 95% CI 0.82–0.93, *p* < 0.001), an RVOT myocardial thickness of 4 mm and above had 90% sensitivity and 87% specificity (AUC: 0.93 95% CI 0.87–99, *p* < 0.001), and a maximal right/left ventricle diameter ratio of 0.91 and above showed 90% sensitivity and 92% specificity (AUC: 0.94 95% CI 0.89–0.99, *p* < 0.001) to predict one-year mortality. A multivariate analysis also demonstrated that RVOT myocardial thickness and maximum RV/LV diameter ratio are independent predictors of mortality.

The current guidelines for staging AS include considerations of aortic valve anatomy, valve haemodynamics, left ventricular anatomy and function, and symptoms [11]. However, this staging does not predict the prognosis, mortality, or potential complications following treatment.

Many parameters have been studied to determine the long-term prognosis in AS. BNP levels, stress tests, and echocardiographic parameters (strain rate, left ventricle mechanical dyssynchrony) have been found to be associated with long-term mortality, even in asymptomatic patients [12,13,14,15,16]. Nevertheless, current guidelines recommend the assessment of LV function only through EF measurement via echocardiographic parameters [3,11]. However, these studies largely focus on LV function as a prognostic indicator in aortic stenosis patients and are primarily conducted on sAVR patients. However, it is known that periprocedural pulmonary hypertension and tricuspid valve insufficiency also have prognostic value in the TAVI patient group [17,18]. Although no statistically significant relationship was found in our study, the female gender was associated with lower mortality in the patient group undergoing TAVI [19].

In the study by Genereux et al., patients undergoing sAVR were classified based on cardiac damage to determine prognosis. The classification was as follows: Stage 0 indicated no cardiac damage; stage 1 indicated left ventricular damage; stage 2 indicated left atrial or mitral valve damage; stage 3 indicated pulmonary vascular or tricuspid valve damage; and stage 4 indicated right ventricular (RV) damage. In the trial, cardiac damage was shown to be one of the strongest predictors of 1-year mortality, with an adjusted mortality hazard of nearly 1.45 with each increase in stage [4]. The study had significant findings. Primarily, it demonstrated that right ventricular dysfunction increases mortality more than left ventricular dysfunction. Although current guidelines recommend comprehensive cardiac imaging prior to TAVI, right ventricular function is often under-recognized in both risk stratification and clinical decision making. Similarly, in our study, greater right ventricular dilation and hypertrophy, as well as pulmonary artery dilation, were observed in the group with increased one-year mortality. When considering both the study by Genereux et al. [4] and our study together, it is concluded that RV evaluation before both surgery and TAVI is important to determine the prognosis and risk. The study by Genereux et al. [4] included a patient cohort undergoing sAVR. Considering that the same disease was treated with different methods, the results of this study may be projected to the group undergoing TAVI. However, TAVI is generally offered to older and higher-risk patients, and findings from sAVR cohorts may not be directly extrapolable to TAVI recipients without further validation, and scientific data on this topic remain extremely limited. Our study demonstrates that right ventricular dilation and hypertrophy, along with pulmonary hypertension indicators like PA dilation, are associated with increased mortality in patients with AS undergoing TAVI. Furthermore, in the study conducted by Genereux et al., cardiac damage was assessed using echocardiography. Echocardiography is subjective and has several limitations, particularly in demonstrating RV dysfunction. In our study, cardiac damage was investigated using CT. Compared to echocardiography, CT provides more objective and reproducible measurements, leading to more consistent results. Although CT has limitations, such as radiation and contrast exposure and availability, it will be beneficial in this patient group because it is routinely performed before TAVI.

Increased right ventricular outflow tract myocardial thickness and pulmonary artery diameter are indicators of pulmonary hypertension on CT [8]. Chronic pulmonary hypertension leads to right ventricular hypertrophy, and a right ventricular myocardial thickness exceeding 4 mm is considered indicative of right ventricular hypertrophy [20]. In our study, an RVOT thickness of 4 mm or more predicted one-year mortality with 90% sensitivity and 87% specificity. A pulmonary artery diameter exceeding 29 mm is also indicative of pulmonary hypertension [21]. In our study, a PA diameter of 30.5 mm or more had 78% sensitivity and 82% specificity (AUC: 0.87, 95% CI 0.82–0.93, *p* < 0.001). These patients were classified as stage 3 in Genereux’s classification [4]. It was noted that the mortality rate for these patients was higher compared to stage 0, 1, and 2 patients. Similarly, in our study, pulmonary artery diameter and right ventricular hypertrophy were identified as predictors of mortality.

In acute pulmonary embolism, numerous CT parameters have been investigated as predictors of mortality, with the RV/LV diameter ratio identified as a significant predictor. In acute pulmonary embolism, a four-chamber RV/LV diameter ratio greater than 1 predicted 30-day mortality with 85.0% sensitivity and 39.6% specificity [9]. This group of patients is classified as stage 4 in Genereux’s classification and represents the cohort with the highest mortality rate [4]. In our study, a right/left ventricle diameter ratio of 0.91 and above demonstrated 90% sensitivity and 92% specificity (AUC: 0.94, 95% CI 0.89–0.99, *p* < 0.001) for predicting one-year mortality. Additionally, multivariate analysis identified it as the most significant parameter for predicting one-year mortality.

In the OCEAN TAVI registry, the relationship between pulmonary hypertension and 2-year mortality was investigated in the cohort of patients undergoing TAVI [18]. Periprocedural echocardiography was used to assess pre- and post-procedural pulmonary hypertension, with sPAP > 60 mmHg considered as indicative of PHT. The presence of pre-procedural PHT, residual PHT post-procedure, or newly developed PHT post-procedure was associated with increased mortality (*p* < 0.001) [18]. Pre-procedural moderate-to-severe tricuspid regurgitation was also associated with residual PHT. In our study, similarly, pre-procedural elevated sPAP and tricuspid regurgitation were found to be associated with increased one-year mortality. Additionally, these results were objectively supported by CT findings.

In the echocardiographic study conducted by Jongh et al., the presence of pre-procedural right ventricular dysfunction and tricuspid insufficiency was associated with increased post-TAVI mortality [17]. Similarly, our study found that right ventricular dilation observed in echocardiography was linked to increased one-year mortality. The five-year follow-up period of that study is among its greatest strengths. However, the study’s reliance solely on echocardiographic data, the semiquantitative measurement of tricuspid insufficiency, and the subjective and variable nature of the fractional area change (FAC) for assessing RV function are among its limitations. In our study, a correlation between right ventricular dilation and mortality was objectively demonstrated through both echocardiography and CT. Furthermore, we provided objective evidence of the relationship between right ventricular myocardial thickness and dilation on CT and mortality.

The Euroscore 2 is a scoring system developed for risk assessment prior to major cardiac surgery and comprises clinical and echocardiographic parameters [22]. Current guidelines recommend it for determining treatment in symptomatic aortic stenosis [3,11]. In our study population, the median Euroscore 2 was calculated as 12, indicating a very high-risk group for major cardiac surgery. Our study found that the pre-procedural scores were similar between the groups with and without one-year follow-up mortality. This can be explained by the different primary endpoints. The primary endpoint of the Euroscore 2 study is in-hospital mortality, and there is no data regarding its prediction of one-year mortality. In our study, the primary endpoint was one-year mortality, and no separate analysis was performed for in-hospital deaths. Additionally, our study is significant as it shows that even in groups with the same Euroscore 2 the group with right ventricular dysfunction had a higher one-year mortality rate.

This study has several limitations. The lack of BNP values for the patients and the absence of baseline right ventricular catheterisation are additional constraints. Another limitation is the lack of follow-up on how the right ventricular damage indicators observed on pre-procedural CT scans changed in the group without one-year mortality. Since patients with good left ventricular systolic function were included in the study, the effect of left ventricular function could not be demonstrated in this study.

In conclusion, consistent with the literature, our study demonstrated that right ventricular damage indicators identified via CT (PA dilation, RVOT thickness, RV dilation) are strong predictors of one-year mortality in patients undergoing TAVI for AS (*p* < 0.001). Before the TAVI procedure, these parameters, that can be easily measured by the operator by using CT, can help determine which patients have a higher mortality risk and ensure the procedure is performed with appropriate caution. To our knowledge, this is among the first studies to investigate the prognostic value of CT-derived right ventricular parameters in patients undergoing TAVI.

## Figures and Tables

**Table 1:** Basal clinical, laboratory, echocardiographic and tomographic parameters.

	Survival Group (*n*: 142)	Mortality Group (*n*: 42)	*p*
Age (year) Median (IQR)	76 (68–85)	81 (72–90)	0.002
Gender Female sex, *n* (%)	76 (53.5%)	23 (54.7%)	0.94
Coronary artery disease, *n* (%)	102 (71.8%)	36 (85.7%)	0.04
Diabetes, *n* (%)	78 (54.9%)	24 (57.1%)	0.835
Hypertension, *n* (%)	28 (19.9%)	9 (21.4%)	0.177
COPD, *n* (%)	37 (26%)	12 (28.6%)	0.172
Creatinine level Median (IQR)	0.95 (0.5–1.37)	1.10 (0.6–1.6)	0.019
GFR Median (IQR)	71 (32)	52 (33)	<0.001
Albumin Median (IQR)	4.1 (3.5–4.7)	4.1 (3.7–4.5)	0.9
Haemoglobin Median (IQR)	12.1 (8.7–15.5)	11.5 (9.5–14.5)	0.18
WBC Median (IQR)	7.32 (4.5–10.3)	7.0 (4.2–10.2)	0.163
LV EF Median (IQR)	50.0 (35.0–65.0)	50.0 (30.0–65.0)	0.375
Systolic pulmonary artery pressure Median (IQR)	40 (25–65)	45 (30.0–70.0)	0.014
Echocardiographic RV dilatation, n (%)	22 (22.9%)	18 (48.6%)	0.03
Severe Tricuspid Regurgitation, n (%)	17 (11.9%)	9 (21%)	0.008
Euroscore 2 Median (IQR)	12 (6.5–16.5)	12.1 (9.5–16.5)	0.155
PAD (mm) Median (IQR)	25 (15–36)	34.5 (28–43)	<0.001
RVOTMT (mm) Median (IQR)	3.5 (2.5–4.5)	6,4 (4.9–7.2)	<0.001
Maximal RVD/LVD Mean ± SD	0.77 ± 0.12	1.15 ± 0.19	<0.001

COPD: chronic obstructive pulmonary disease, GFR: glomerular filtration rate, IQR: interquartile range, LV EF: left ventricle ejection fraction, LVD: left ventricle diameter, mm: millimeter, PAD: pulmonary artery diameter, RVD: right ventricle diameter, RVOTMT: right ventricle outflow tract myocardium thickness, SD: standard deviation, WBC: white blood cell.

**Table 2:** Multivariate Cox regression analysis.

Variables in the Equation			
	B	Wald	*p*
Age	−0.018	0.302	0.583
Coronary artery disease	−0.929	1.299	0.254
GFR	0.005	0.184	0.668
Systolic pulmonary artery pressure	−0.007	0.206	0.650
Severe tricuspid regurgitation	0.344	0.085	0.771
RV dilatation	−0.362	0.000	0.998
PAD > 30.5 mm	−0.0227	0.146	0.703
RVOTMT > 4 mm	−1.729	4.666	0.031
RVD/LVD ratio > 0.91	−2.326	8.685	0.003

GFR: glomerular filtration rate, LVD: left ventricle diameter, PAD: pulmonary artery diameter, RVD: right ventricle diameter, RVOTMT: right ventricle outflow tract myocardium thickness.

## Data Availability

The data presented in this study are available on request from the corresponding author due to privacy.
